# ‘Everyone needs to be educated’: pupils’ voices on menstrual education

**DOI:** 10.1186/s12978-024-01862-6

**Published:** 2024-08-20

**Authors:** Natalie Brown, Laura J. Forrest, Rebekah Williams, Jessica Piasecki, Georgie Bruinvels

**Affiliations:** 1 School of Sport and Exercise Science, Swansea University, The Bay Campus, Fabian Way, Skewen, Swansea, SA1 8EN UK; 2 School of Health & Life Sciences, University of the West of Scotland, Glasgow, UK; 3 Stride Active CIC, Herefordshire, UK; 4 Sport, Health and Performance Enhancement Research Centre, Nottingham Trent University, Nottingham, UK; 5 Institute of Sport, Exercise and Health, University College London, London, UK

**Keywords:** Menstrual health, Periods, Students, Curriculum

## Abstract

**Background:**

Physical, affective and behavioural symptoms associated with the menstrual cycle are commonplace. Adolescents with problematic symptoms have reported a negative impact on school attendance, behaviour and participation in physical activity. In the United Kingdom, evidence suggests that menstrual health education delivered by teachers focusses on menstrual cycle biology as opposed to management of menstruation and menstrual cycle related symptoms. Through obtaining the pupil voice, this study aimed to understand young people’s perceptions and experiences of menstrual education in schools and their experiences of menstruating whilst at school, including within Physical Education.

**Methods:**

To address the aims, a qualitative descriptive study was conducted. Nine focus groups with female pupils (n = 48; ages 10–15 years) were completed across six school locations in England and Wales, including primary and secondary schools. A range of elements relating to the menstrual cycle, education at school and PE were discussed by pupils.

**Results:**

Four main themes were developed (a) Education preferences, (b) Period positive environments, (c) Personal experiences and (d) Impact on school. Similarities were reflected across focus groups in terms of current (lacking) education, lesson style and preferences, comfort of conversations, lack of school support and impact on school and PE.

**Conclusion:**

The findings highlight the lack of menstrual education received and subsequent impact of menstruation and menstrual-related symptoms in school and PE. The abundance of information requested by pupils highlights the deficit in understanding about how to manage periods in school and remain engaged in physical activity. Schools can utilise information from the current study to; create period positive environments; consider lesson content and format; and explore how to support girls to stay physically active in PE.

## Abstract (highlights)

Female pupils attending primary and secondary schools across England and Wales have shared their perceptions and experiences of school menstrual education, including perceived gaps in their own understanding. Pupils highlighted education was limited and sometimes no lessons were provided. Generally education focussed on the biology of the menstrual cycle rather than lived experiences and management solutions, as requested by pupils. Discussions also explored pupils’ preferred learning style and environment for period education. A negative impact of the menstrual cycle on school was evident, especially within Physical Education (PE). The wider school environment, such as access to toilet facilties was repeatedly raised as an issue across all focus groups. The information collected highlights the lack of menstrual education received and subsequent impact of menstruation and menstrual-related symptoms in school and PE participation. The abundance of information requested by pupils highlights the deficit in information provided to young people to understand and manage periods in school.

## Background

The menstrual cycle is a normal biological process for females [1] which occurs from puberty through adulthood until menopause. Ovarian hormones fluctuate in a cyclical pattern, with ovulation occurring approximately 11–17 days prior to menses [2]. The changes in ovarian hormone levels can result in menstrual cycle-related symptoms and signs. Up to 90% of the female general population experiencing some form of problematic menstrual cycle-related symptoms [3]. While symptoms are very common, and historically normalised, physical, affective and behavioural symptoms and signs associated with the menstrual cycle can have detrimental implications for individuals [4].

School experiences and participation in physical activity and exercise can be affected by menstruation and menstrual cycle-related signs/symptoms in adolescence. These have detrimental effects on quality of life, health, academic and professional performance [5]. In the United States, school teachers perceived (n = 209) student experiences surrounding menstruation to be negative, stressful, embarrassing and often concealed [6]. It was perceived that adverse symptoms caused students to be distracted from learning, with 81% believing menstruation to impact upon student learning [6]. Similarly, a negative effect on school behaviour and participation in Physical Education (PE) was reported by 88% of teachers (n = 694) [7]. This research is supported by recent reports which cite periods as one barrier to physical activity and PE by adolescents in the UK [8]. Given persistent low physical activity levels amongst adolescent girls is a public health concern [9], there is a need to identify the reasons why periods are the most reported barrier to PE to inform future solutions.

Until recently, much of the evidence around girls’ experiences of menstruating and menstrual education in schools has been focussed on low- and middle-income countries [e.g. 10]. Challenges for students from low- and middle-income countries often stem from cultural stigma, taboos and infrastructural barriers including inadequate menstrual hygiene provision [11]. However, less is known about menstrual experiences of girls in developed countries. School students (n = 12) in the United States described multiple challenges when learning about and experiencing menstruation in school [12]. This included limited understanding of the menstrual cycle, lack of access to menstrual products, limited time for changing products and fear of leaking [12]. A similar narrative was identified in Australia from 5007 young women aged 13–25 years, in which menstrual education focused on biology, and overlooked the effect of the menstrual cycle citing it being ‘too little, too late’ [5]. However, perspectives of students are unknown in the United Kingdom. There is limited understanding of young people’s experiences of menstrual education received in school, their perceived knowledge of the menstrual cycle and the consequential impact of periods on their schooling.

The student voice is a developing concept; placing young people as key stakeholders in their own learning, enabling them to be co-creators of educational practice [13]. Involving students in research is considered important in shaping educational changes and reforms [14]. Therefore, through obtaining the pupil voice, this study aimed to understand young people’s perceptions and experiences of menstrual education and menstruating in schools. This included perceived gaps in their own understanding, personal experiences, and levels of comfort when having conversations with teachers, peers and parents. A secondary aim was to explore first-hand how girls felt their periods affected educational learning and participation in PE. In doing so, the findings may inform future educational practices.

## Methods

### Study design

To address the aims of this study and facilitate an in-depth understanding of pupils’ experiences, a qualitative descriptive study was conducted [15, 16]. Interpretive description (ID) [17] methodology was used for this study. ID is a non-categorical, inductive method of identifying relationships and developing conceptual descriptions which can be used in applied settings [17]. ID aims to answer questions about how people experience the world; knowledge is constructed by individuals based on their experiences and interactions with the world. Philosophically, ID is underpinned by ontological and epistemological social constructivism. Specifically, ID assumes there to be multiple constructed realities and that knowledge development is the result of an interaction between the researcher and object of inquiry. ID was deemed appropriate for the current study as no research has examined the experiences of menstrual education in United Kingdom schools. Furthermore, the applied nature of ID matched the overall desire of this research to produce practically useful results.

The use of focus groups with a moderator (NB) best aligned with the requirements of the study. The purpose was to gain an in-depth understanding of the topic from pupils’ experiences through a detailed discussion. The unique benefit gained from focus groups is the opportunity to collect rich information using group discussions and interactions [18]. Focus groups can encourage participation from people who are less inclined to be interviewed individually, particularly around sensitive topics [18] such as menstruation.

### Participants

Following receipt of institutional ethics approval (NB_02-11-21d), convenience sampling was employed, supported by Youth Sport Trust. Teachers were contacted via email and those interested in participating replied to the lead researcher with an expression of interest. The lead researcher followed up directly with the teachers to share participant information and organise a suitable time to complete data collection. Teachers facilitated the recruitment of participants, sharing the participant information sheet, consent form and assent form. Parental consent and participant assent was required before focus groups began. Where possible, purposeful sampling was applied to recruit a mix of urban and rural schools, incorporating a range of socio-economic demographics from a minimum of two cities. Five schools were initially recruited, with one further school recruited to reach data saturation [19]. Inclusion criteria defined schools in the United Kingdom, participants who were observed and noted as female at birth from years 6 to 10 (ages 10–15) in England and Wales, or equivalent. Focus groups were planned according to school year groups, year 6, year 7–8 and year 9–10. This was for logistical purposes i.e. year 6 participants attend different schools to years 7–10 in England/Wales, or to differentiate age-associated differences i.e. secondary school year groups divided into two separate age groups to acknowledge different experiences of their menstrual cycle. Participants were not required to have reached menarche. The experiences and education received by those yet to start menstruating was important to understand if they felt supported and what information they would like to receive to feel prepared for menstruation.

### Data collection

All focus groups were completed June-July 2022. Prior to the focus groups commencing, consent was re-established, and voluntary participation was emphasized with the ability to withdraw at any point (no incentives were provided). Participants were reminded there were not any correct or incorrect answers. The focus group guide began by exploring menstrual education in schools, including current content, educational approaches and what education the participants would like to change or receive. Participants were then asked about their comfort levels when conversing on periods in a school environment, before culminating with a focus on periods when participating in physical activity (Table 1). The first author (NB) was responsible for moderating all focus groups.

**Table 1:** Topic areas and key content covered in participant focus groups with example questions

Topic area	Content	Example questions
Menstrual education received at school	Receipt of education, year received education, lesson content, who delivered lessons, what format were lessons, preference of learning style	Who delivered the lessons you’ve had? How did you find the information they shared?
Comfort of the topic	Learning at school, talking to teachers, talking to family/friends	Who do you talk to about your period? Why do you talk to that person and what stops you talking to others?
Support	Period products, teachers, information	Have you received any information about coping with your period/symptoms generally in school? What about for PE?
Personal experiences	Symptoms, manging periods/symptoms at school	How do you feel about being on your period whilst at school? Does it affect you at all e,g, days off, taking part in PE, academically?

To prevent biases introduced by group dynamics and the influence of dominant participants within focus groups, the moderator ensured all participants were able to contribute by selecting those who had not spoken much to continue if multiple participants initiated responses at the same time. Also, it is recognised that biases and assumptions can influence the framing of the questions. However, questions were developed by a multidisciplinary team of experts, with expertise in qualitative research to avoid this. In addition, the questions were developed based on an extensive review of the existing literature along with key stakeholder input (i.e. teachers and young people) provided feedback on the wording of the questions. These processes were aimed at ensuring the questions were relevant, clear, and capable of eliciting in-depth and meaningful responses from the participants. A pilot focus group was completed with pupils in advance of data collection to further support this process.

### Data analysis

Each focus group was audio-recorded and transcribed by a professional transcribing service. Transcripts were checked for accuracy and any personal identifying information was removed. Each transcript was analysed by the first author (NB) using qualitative data analysis procedures recommended by Miles, Huberman & Saldana [20]. Data reduction was completed using three stages of coding. Firstly, descriptive codes were assigned to the data to identify raw data themes.This allowed for interpretive codes to be generated which were then grouped into more abstract concepts. Next, pattern codes were identified which recognised relationships between interpretative codes. The results were produced by researchers working as a team. The second author (LF) questioned the analysis and asked for explanations and justifications for the codes produced. Taking one sub-theme as an example, ‘lesson style and approach’, this grouped codes of mixed/single sex lessons, teacher preference, teacher sex. The next analytic step involved a second author (LF), questioning raw data themes. Continuing the above example, lesson style and approach was grouped into the main theme of Education preferences. This resulted in some reorganization of the grouping of themes but not of the coding itself; for instance ‘Period Positive Environment’ grouped school support and communication which previously sat within different main themes of Education Preferences and Personal Experiences, which after discussion it improved clarity of the results. The final phase of analysis was the writing of the results section as this is viewed as part of the analysis in qualitative research.

## Results

Nine focus groups were completed across six school locations (Table 2). Each focus group had 4–6 participants, divided into year 6, years 7–8 and years 9–10 groups. All focus groups lasted between 35 and 60 min (m = 43.95). A word document containing approximately 62,420 words of transcribed text was analysed.

**Table 2:** Participant focus group characteristics

School location	Year Groups	Total number of participants	Number of focus groups	Geographic area	Percentage eligble for free school meals	Gender of entry
London (L1)	7–8 and 9–10	9	2	Urban	25.4	Girls
Derby (L2)	7 and 9	8	2	Urban	22.5	Mixed
Herefordshire (L3)	8 and 10	10	2	Rural	21.1	Mixed
Kent (L4)	8	4	1	Urban	25.2	Mixed
Torfaen (L5)	7–8 and 9	12	2	Rural	Not recorded	Mixed
Sheffield (L6)	6	5	1	Urban	9	Mixed

A range of factors relating to the menstrual education and PE were discussed by participants. Similarities aligned to current education and preferences, comfort of conversations, lack of school support and impact on PE were reflected across focus groups. Through analysis, four main themes were developed a) Education preferences, b) Period positive environment c) Personal experiences d) Impact on school. Twelve sub-themes emerged from the main themes (Table 3). Each main theme is discussed below.

**Table 3:** Main and sub-themes from the focus groups

Main Theme	Sub-theme	Illustrative Quotes
Education preferences	Lesson style and approach Lesson content and delivery Lesson timing Education for non-menstruators Physical activity and education	Boys and girls were split up to learn about the female body parts (L1, y7-8) They just taught us how to use pads and tampons (L3, y10) We learnt about them [periods] in year 6 (L2, y9) It would be good if boys were educated as well, to make it less awkward (L4, y8) They don’t [provide information], they like you to deal with it [menstrual cycle symptoms] and just get on with it [PE] (L3, y10)
Period positive environment	Awareness School support External support Communication comfort and confidence	People don’t have the same period pain as other people…it’s different for every person (L3, y8) I have come to realise the school doesn’t have much sympathy when you are on your period. It’s very like oh get on and deal with it (L1, y9-10) I spoke to some other older girls…and some were like “we will use extra, so we’ll use a tampon and a pad when we’re doing exercise just to be doubly cautious” (L3, y8) It depends on the relationship that you have with your teacher because if you don’t really like that teacher or you don’t teally speak to that teacher, then you are going to feel more uncomfortable (L3, y8)
Personal experiences	Symptoms Negative experiences Management	Pain, I get it in my stomach and my back. I feel I change when I have my period and get angry or upset. (L3, y8) One time in year seven I opened my bag and it [menstrual product] flew out and everyone was just laughing and I was so embarrassed (L1, y9-10) When we did this This Girl Can Festival, I was on [menstruating] and it really helped (L3, y8)
Impact on school	General school (e.g. attendance, engagement) Physical Education	It affects how much I attend in lessons. Say, how much I get distracted…that’s gonna affect my learning and stuff. (L3, y8) I’ve had to take time off from school as well because it’s so painful (L3, y10) Just before we were about to go swimming and I got really annoyed because I was just like “I don’t really want to go in the pool now because I have a fear of leaking” so I had to sit out the whole time (L3, y8)

### Education Preferences

Participants shared different experiences of menstrual education received at school. Participants often explained their preferences to receive education separately from boys, to increase levels of comfort and reduce feelings of being judged or embarrassed. Where menstrual education was taught in mixed-sex classes, some participants shared how boys’ responses affected the content and reduced the opportunity for conversation and questions.

However, participants clearly voiced that boys should also receive education on the menstrual cycle to increase their understanding and compassion. This could be in separate lessons or initially separate before rejoining a mixed-sex class; ‘If boys learn about it first, for two lessons, and then we had the lesson with them it would probably be alright’(L4, y8). If lessons were mixed, which some participants were comfortable with, participants requested an additional opportunity with just girls to ask questions.

Although it may be more comfortable to have like a lesson with just females, the boys need to learn about it more because it is like a serious topic. They just joke about it’s the most embarrassing thing and it’s horrible. (L5, y9)

Irrespective of sexes present within classes, smaller classes were requested to facilitate positive conversations compared to large classes which were described as ‘awkward’;

I think if it’s in a big class you feel more awkward trying to say what you’re trying to say than if you’re in a small group. (L3, y10)

Participants perceived teachers were capable of providing menstrual cycle education; ‘I don’t mind teachers telling us about it because it’s their job, they’re teachers’ (L3, y8). Although in some instances there was a preference to learn about the menstrual cycle from a medical expert. If teachers were delivering the education, their level of confidence was important: ‘Because of her [teacher] confidence, that helps us gain confidence about asking questions’(L3, y8).

The preference was to receive education from female teachers due to their lived-experiences and perception of being more understanding than male teachers;

It would be better if a female teacher [delivered lessons] because they have periods. I had a male biology teacher, and you could just tell the male teacher didn’t really know what to say, he was just reading what was on the board. (L4, y8)

Participants frequently disclosed they only had one menstrual education lesson at school and the content delivered was often perceived to not be supportive or useful. In some instances, menstrual education was completely absent which was exacerbated during the COVID pandemic (March 2020-July 2021). The timing of menstrual education was frequently reported to be too late; ‘I think for younger years, like Year 7 or Year 8, it would be quite helpful’ (L3, y10). Furthermore, a lack of information was received prior to reaching menarche; ‘I think before I got my period I was so scared because they never told us’. (L5, y7-8).

I feel like there’s a lot of people who don’t know what it [menstruation] is. But I think it should have been a lot earlier, like that’s a problem, everyone needs to be educated about everything about female puberty, like everything. (L6, y6)

Commonly, information on what to expect and what to do on your first period was delivered in primary school (years 5–6), along with information on some period products (tampons and pads). Information on reproduction was sometimes received during biology lessons in secondary school, alongside symptoms (period cramps, mood swings, body changes). However, this focussed on educational content of the Biology curriculum rather than considering the impact on individuals. Participants also reported limited education and information on the causes of symptoms, how to cope with different symptoms, and recognising and dealing with irregularities. Even more so was the complete absence of any education on how to manage periods during PE. Participants felt teachers approached menstrual education lessons as ‘a tick box’ exercise with perceptions that teachers do not value the importance of the lessons; ‘ok we have to get through these slides so we can move onto the next thing’ (L1, y9-10). Lessons were reported to lack detail of personal/lived experiences and management of symptoms. Table 4 summarises content and information requested by participants.

**Table 4:** Lesson format, delivery, information and supported requested by pupils relating to menstrual education and school for a period positive environment

Lesson format and delivery	Requested school support
Mixed-sex classes with opportunity for girls only class after OR boys and girls separate and then a mixed-sex lesson after	Timing of delivery useful for pupils rather than convenient in the school year
Frequent lessons to recap or talk/ask questions	Avoid lessons being a ‘tick box’ exercise
Smaller class size	Aim to dispel myths e.g. not being able to do handstands whilst on periods!
Female teacher to deliver lessons	Access to toilets during lessons
Teachers to be confident delivering	Access to period products in toilets
Lessons delivered at a younger age (year 10 too late)	Male teachers having increased understanding
Anatomy diagram where the reproductive system is and what this includes e.g. where does a tampon go and how to insert a menstrual cup	Teachers to receive education
PE lessons to include slower exercise or options e.g. yoga across all school years	Empathy from PE teachers
Information requested	
How to know when your periods start	Teachers listen and acknowledge individual differences
Why do females menstruate and what happens throughout the cycle	Develop an open culture and normalising conversations amongst boys and girls
How much blood is lost and how to manage heavy bleeding	Peer support
Menstrual irregularities, what is normal and abnormal including menstrual dysfunction	Boys to receive lessons
Causes of absent or missed periods	Include parents in increased knowledge and support of the menstrual cycle
Causes of different colour of period blood	
Causes of menstrual cramps and management strategies including exercise	
Causes of mood swings	
How to track the menstrual cycle	
Understanding of different menstrual products	
Management strategies for periods and staying physically active	
Who to talk to about the menstrual cycle and any issues	
Other management strategies available such as diet and medication	

Participants perceived a lack of education and understanding of the menstrual cycle by non-menstruators in schools. Participants reported that male teachers particularly lacked knowledge, confidence and displayed feelings of discomfort during conversations; ‘there are certain male teachers, but there are only some that really understand and stuff’(L3, y8). However, some participants did describe positive experiences of speaking to male teachers, or of the lessons taught by a male teacher:

He was confident speaking about it even though it hasn’t happened to him. He was still confident speaking which I think made the girls a bit more…he knew a lot about it surprisingly. (L3, y8)

The perception and impact of boys’ attitudes towards the subject was prominent. Participants shared how boys’ lack of understanding, asking uncomfortable questions and laughing or making fun of the topic had a negative effect on girls; ‘Boys shouldn’t be teasing girls for something that is not their fault and it’s natural’ (L5, y9). This was further emphasised by comments such as:

The boys in our school they thought like you could control when you had your period. Like you could be like, I want my period now and I don’t want my period now and yeah, I’m just going to make it stop (L2, y7-8)

Because they make fun of it [menstruation], some people don’t change [period products] in school and that’s real bad. (L5, y7-8)

Some participants suggested ‘Maybe if the teachers, like the male teachers got to know a bit more about it, maybe that would be a bit better’ (L6, y6).

Participants expressed desired changes in PE, linked to activities and exercises. There was a consensus for greater flexibility in activity choices if experiencing menstrual cramps, with a preference for slow movement and exercise such as yoga, ‘if we did like a slower form of exercise’ (L1, y9) or to be involved in a different capacity such as coaching. Year 10 participants felt they had more choice, but suggested this should be throughout all school years, given not all girls began their periods in the same school year. The opinion within this group was quite clear, highlighting the need for options. Generally, participants wanted to know how to manage their menstrual cycle to facilitate participation in PE, or have the option to participate in a more appropriate activity for their symptoms.

It might be really encouraging for younger years to choose because if their influence at the age of 11 or 12 that doing sports on their periods is a really horrible thing they will take that with them as they get older which is not a good thing but if they choose their sports then enjoy that and then that would be really good thing (L1, y9-10)

Education was needed on how to manage periods whilst exercising, ‘we haven’t been told. Not by parents, not by teachers’ (L5, y7-8) and requested support to use exercise to help manage symptoms;

Rather than saying sit out of PE they [teachers] can give the girls who are on their periods like time to do exercise that helps with cramps or reduces pain. (L1, y9-10)

### Period positive environment

Participants demonstrated a lack of awareness relating to many aspects of the menstrual cycle within school and wider society. For example, participants lacked anatomical awareness ‘so where is your uterus, is it in your stomach?’(L2, y7), the biology of the menstrual cycle and often had misunderstandings around what is deemend a normal menstrual cycle, ‘I always thought like periods were like 24/7 like every day, all the time. That’s why I was like terrified’ (L5, y9) and when menses generally commence. There were also gaps in understanding around management of the menstrual cycle and menstruation from both a period product perspective and causes and management of symtpoms ‘why do you have cramps, like, what causes them?’ (L2, y7). Furthermore, there was a limited awareness of when and where to receive support in relation to their menstrual cycle.

Two participants indicated increased knowledge of the menstrual cycle; one participant had an awareness of energy intake and missed periods ‘not drinking and not eating much like can cause irregular periods’ (L5, y9). However, this level of understanding was rare.

It was consistently reported that some aspects of the school culture and environment were unsupportive for individuals menstruating. Some teachers were reported to lack awareness and perceived to not listen to individuals’ experiences of menstruation, ‘You just feel like they [teachers] don’t understand. It puts you in a worse mood knowing they [teachers] are not listening’ (L3, y10). Frequently this was associated with PE, ‘so basically if you had a period and you did PE like they [teachers] are not sympathetic like they don’t care’ (L1, y9-10).

Access to toilets was discussed in all focus groups with many participants revealing that this was limited or not possible during lessons:

In lessons if you need to use the bathroom to change your pad or something, going to the toilet in lessons is very difficult and unless you specifically say I am on my period which for some people is embarrassing then it’s very difficult to go to the toilet during lessons (L1, y9-10).

Mixed or unisex toilets caused unease due to comments from peers when changing period products;

The toilets, like the girls and boys mixed together, like sometimes I don’t feel comfortable, like if you have a pad and you are taking it out sometimes they will ask you what you are doing…it feels kinda weird. (L2, y7)

Despite this, PE lessons were deemed most difficult by some. Participants shared how some PE teachers lacked empathy or prevented access toilets whilst out on the field during lessons: ‘they don’t really let you go to the toilet so it can be really stressful’ (L4, y8).

All focus groups reported period products were available in their schools, yet there were varying degrees of embarrassment associated with accessing products. Some participants felt too embarrassed to ask a teacher or school reception staff where they were stored. However, participants did demonstrate an understanding as to why products were not supplied in toilets; ‘I think that if you put pads and tampons in the bathroom, some people might take them and be stupid with them’ (L3, y8). Many participants requested changes to where menstrual products were stored and better access to toilets as two important factors that would improve experiences whilst at school (Table 4).

Participants did share positive examples of supportive environments that had been created within schools; ‘There’s an awareness to it [menstruation]; there’s little poster things telling you about the period products and ask the nurse if you need them’ (L2, y9). Experiences of specific supportive teachers were also shared;

Well our PE teacher is quite um, well he understands when if we do have our period then he says if there is a spillage or anything then you can go straight to the toilet its fine, but he understands that some activities will be harder for us because of cramps. (L6, y6)

Additional education at school was requested (Table 4), for instance there was a unanimous request for management strategies such as, ‘how to cope if you have cramps; diet or exercise that can help prevent it’ (L3, y8) along with ‘tips to deal with it [menstruation], like from the older female pupils like they know more tips about it’ (L6, y6) and having a more open culture in school:

Maybe instead of having certain times that we do this [menstrual education] we should like every week [have] an open conversation where people can share their thoughts on it [menstruation] (L6, y6)

Uniform and PE kit were discussed across the focus groups with mixed experiences; dark uniform was preferred and there were negative experiences of skirts for PE kit; ‘don’t get me started with wearing a skirt during PE. I would just quit wearing those skirts’ (L1, y9-10). Whereas there was a positive response where PE kit had been changed;

I really liked when the leggings first came out because it makes me feel more comfortable when I’m doing PE because if you leak, it’s not really noticeable compared to when you’re wearing a skirt or shorts. (L3, y8)

It became apparent across the groups that choice was essential;

I think it’s good of the school to at least not make you wear skirts, like you can wear joggers, you can wear shorts and leggings, there’s a variety of things you can wear. (L2, y7)

Participants reported external support to ‘fill the gaps’ in their knowledge of the menstrual cycle, where this had not been supplied in school. Most frequently, social media (Instagram, TikTok) and the internet were identified as sources of information (YouTube) ‘I didn’t find it out from school I found out from YouTube’ (L6, y6):

This thing that I saw on TikTok and it’s called Polycystic Ovaries that can affect your period, right? I have never learned about that in school, and I learned [it] on TikTok and it’s really bad. (L1, y9-10)

Others shared that family, especially mothers, provided menstrual cycle-related information and education at home.

Levels of comfort and confidence when conversing on the topic were variable amongst participants and dependent to whom they were speaking to. Participants tended to have conversations with mothers, family members and friends. In some instances, the preference was to speak to teachers over parent/guardians. However, this was not consistent across all participants; some highlighted discomfort and consequently did not talk to anyone due to feeling too uncomfortable or scared; ‘I wouldn’t speak to my teachers about it’ (L1, y7-8).

Different factors were presented that affected comfort of conversation, for example school environments: ‘it just wouldn’t feel appropriate [to talk] because my class is quite a handful and so like they’re [teachers] probably concentrating on other things’ (L2, y7). Gender and personal experience was summarised by another participant as a factor affecting comfort of conversation; ‘I don’t really want to talk to a male teacher because it’s a kind of thing like they wouldn’t know much about’ (L2, y7) along with teacher openness; ‘they [teachers] never talk about it with us so I would just rather not’ (L1, y9-10). Perceptions of teacher awkwardness was another factor reported; ‘some teachers can be a bit awkward, I mentioned it to a boy teacher and he looked very panicked’ (L4, y8), whilst additional factors such as familiarity with the teacher and surroundings were shared. Initiation of conversation by another person was described as an influencing factor, for example, ‘if someone talks about it [menstruation] first, then I’ll talk to them back to give them advice or something and then we’ll get into a conversation about it, but I wouldn’t choose to start the conversation’ (L3, y8). One participant summarised:

Personally, I think that the reason why most teachers are like that is because we weren’t open to them in the first place because I think maybe because like if we talked to them sooner or we told them a bit more they would be a bit more understanding (L6, y6)

### Personal experiences

Participants reported a wide range of menstrual-related symptoms experienced, but a lack of education on management strategies to use whilst in school. Physical symptoms (e.g. menstrual cramps) and psychological symptoms (e.g. mood swings) were reported by participants across all focus groups. It was consistent that the physical symptom of bleeding was associated with negative experiences at school. Participants reported their worry that others can see pads or know they are on their period; ‘at school I’m conscious of anyone, if you can see it. If they can tell you’re on [menstruating]’ (L2, y9). Getting changed in PE exacerbated this; ‘I don’t like getting changed in PE in case anyone sees anything’ (L2, y9). A period starting during a lesson and the fear of leaking in lessons was reported as being shameful and a nuisance. Words such as ‘embarrassing’, ‘suffering’, ‘inconvenience’ and ‘annoying’ were used to describe experiences of periods whilst at school. This was enhanced by the fear of boys' reactions, and occasionally girls', making fun of them. The worry of irregular periods and bleeding unexpectedly was also evident.

Alongside the emotional aspects of managing at school, the physical symptoms were reported: ‘we all hate cramps’ (L6, y6). Suggestions such as using heat/hot water bottle and exercise were shared as symptom management strategies. Sometimes the lack of management was related to limited knowledge of what consistutes a healthy menstrual cycle and pain experienced, access to products and confidence to have conversations, as summarised by one individual’s negative experience;

The first time I had my period was when I was wearing a skort and it was PE when I got my first period and I had to go into the bathroom but because I didn’t know a lot about it, I didn’t have any pads or anything so I had to use tissue and I was really scared cause I hadn’t known much about it and I didn’t really know what to do, so I didn’t tell anyone. I didn’t tell my mum. I used a pad when I got home in my bathroom. I just did that until I brought up the courage to tell my mum that had happened and that’s when we got the leggings because I felt more comfortable (L3, y8)

Tracking of menstrual cycles, particularly using mobile applications, was discussed as a management strategy:

I've got an app on my phone that I use. I think since using it... because before I used to get quite scared that like I was going to like start when I didn't have stuff on me, but now I know like a week before that I need to take stuff in. (L3, y10)

One participant shared the benefit gained from a supportive and open environment when participating in community sport, which she applied to PE lessons also; ‘I’m a gymnast myself, so like the gym are really supportive…so I’ve sort of learnt how to cope with it [menstruation]’ (L5, y9).

### Impact on school

Whilst some participants acknowledged the menstrual cycle does not affect them in school, the majority of participants discussed the negative impact menstrual-related symptoms can have on learning, engagement and attendance. Generally, participants reported that the first two days of their period were the worst for affecting their learning and engagement in lessons. Being distracted by stomach pain or the worry of leaking were discussed. Participants talked about Difficulties managing emotions and concentration, Low motivation, Increased tiredness and Negative impact on school attendance and behaviour. School attendance was affected, with reports of menstrual cramps causing missed school days (Table 3). Whilst another participant mentioned ‘we had SATs (Standard Assessment Tests) recently so if anybody had their period then that would be like really annoying’ (L6, y6).

However, participants reported PE was the most affected aspect of school.

PE on my period, it’s awful. The pain and uncomfortable, you feel like you are going to leak and stuff (L4, y8)

Menstrual cramps affected participation in PE, with preferences not to exercise or take part in PE whilst menstruating. Timing of PE in relation to menstruation was a consideration for some participants; ‘if it’s the first two days it’s horrible to do it [PE]’ (L2, y9) and the type of activity undertaken also being noted as a barrier by some:

We were jumping and everything and I only jumped once because of how bad it [menstrual pain] was. I feel like I don’t want to do it because of how bad it is’. (L3, y8)

It affects my ability to do some activities. For example, you can’t do swimming, I can’t, I love running and I can’t do running at break times because it hurts bad. (L6, y6)

Leaking was also mentioned as a fear that impacted participation in PE; the following experience summarises that of many participants:

The first time I put my leggings on, and I immediately bled through by accident, so I didn’t do PE and I was like really embarrassed’. (L5, y9)

Menstrual-related breast pain was also reported to affect participation in PE, ‘period boobs, where it’s just like for example when you’re running, and it just hurts so much’ (L5, y9). Both the fear of leaking and pain were attributing factors to the type of exercise participants were more likely to take part in (e.g. yoga) compared to those they would avoid. The activities reported to avoid included running and jumping, gymnastics and swimming. Unfortunately, even participants engaged in swimming felt they were unable to and had no choice. Conversely, in other activities, participants requested having the option to take part in a different capacity, such as coaching.

Menstrual related symptoms such as low mood and ‘feeling extra tired’ (L2, y9) also affected participation in PE. This was reported to influence signing up for competitive events such as sports day, ‘you could have signed up for running the week before that, you wouldn’t know you would be due on [menstruating] you get to the day and don’t want to do it’ (L4, y8). However, some participants’ enjoyment for PE negated menstrual cycle symptoms and, whilst others were not affected by menstrual cycle-related symptoms and therefore participated in PE so did not perceive periods to stop them.

## Discussion

The intention was to engage with young people to understand their experiences of menstrual education and impact of periods on participation in PE as a subtopic of school experiences. Overall, the findings of this study highlight the expansive impact menstruation and menstrual cycle-related symptoms has on school and PE participation. This is particularly linked to lack of education received in school, negative experiences of menstruating at school and lack of comfort talking to teachers, especially those who were perceived to lack confidence/knowledge on the topic. The abundance of information requested by pupils highlights the likely deficit in education provided to pupils to understand and manage periods in school and remain physically active, specifically in PE.

### Schools offer a platform for education

Schools can play an important role in developing health literacy because of curriculum requirements on personal development and the large proportion of time children spend in education [21]. Menstrual health is integral to improving health literacy and can have a direct impact on young peoples’ quality of life, health, academic and professional performance [5]. However, as identified from our current findings, adolescents within the focus groups lacked understanding of the menstrual cycle. Research continues to highlight the gaps in school curriculum, missing topics such as menstrual dysfunction and health literacy surrounding the cycle [22]. These findings are supported by previous research where adolescents were unable to assess whether their period was typical [23]. In the case of high-income countries, young females have reported a lack of support from schools in relation to menstruation [24]. Studies have highlighted the need for a stronger provision of all school based menstrual education [23, 25].

Our previous research, which surveyed teachers, outlined the lack of menstrual education provided in schools across the United Kingdom. Only 63% (n = 498) of primary and secondary teachers reported menstrual education was provided; when it was provided, the primary focus was to teach the biology of the menstrual cycle (56%) or provision of menstrual products (40%) [7]. The findings of the present study reveal similar experiences from pupils, providing parallel views between the two groups. Pupils reported limited education, in some cases receiving no information at all from school; instead gaining information and support externally via parents or social media/internet. Where information was provided in schools it was factual, focussed on what happens and was perceived as a ‘tick box’ lesson. This left girls with a lack of education on lived experiences, finite awareness of menstrual irregularities, including anovulatory cycles, or management solutions. Furthermore, they had a complete absence of support on staying active whilst menstruating. This is consistent with findings in the United States where participants (n = 12, aged 12–16 years) described challenges faced when learning about and experiencing menstruation in school [12]. Further research in Spain identified how 36% of participants (n = 2578) did not receive information on menstruation prior to menarche or on how to manage it [26]. This pattern continues in other areas such as Australia [5]. This can result in poor menstrual health and long-term negative health consequences for some individuals within global societies. However, research in Australia has shown that a school based ovulatory menstrual health program can improve menstrual health literacy [27].

It is recommended that menstrual education, beyond reproductive biology, should be integrated into school curricula. More importantly, this education should be provided for everyone, including those who do not menstruate [26]. This message was strongly emphasized by participants in the current study. Listening to the voices of those who menstruate, fostering a more supportive environment and educating teachers on how to positively address student menstruation experiences is required across all of society [6]. Generally pupils requested lessons to be delivered by female teachers, with lived experiences due to perceptions of increased understanding. However it is important to note that having the lived experience of menstruation is not a sufficient qualification to educate others about it, and instead all teachers should be provided with training to increase their knowledge and understanding to best support pupils in schools.

### Appropriate facilities

Unlike research in the United States [12], pupils in the current study reported awareness of access to period products in their schools. However, the location of products and thus the discomfort in requesting period products from teachers presented a larger barrier to access.

There are multiple implications of restricting access to toilets in school. In relation to menstruation, previous research has identified how the prohibition of toilet breaks prevented the management of bleeding and became associated with distress of leaking [28, 29]. This is consistent with findings in the current study. Across all focus groups, pupils consistently discussed the concern of being unable to access toilets and the related fear of leaking during class. This resulted in pupils feeling distracted during lessons and feared standing up at the end of class. A further toilet issue was raised; pupils feared changing period products, especially in unisex toilets. As a consequence pupils queued to use female-only toilets or avoided changing period products throughout the school day, which can negatively affect health and wellbeing. Schools need to consider the negative impact of unisex toilets and restricted toilet access; both have implications for management of menstruation and creating a period positive environment.

### Specific PE considerations

Persistent low physical activity levels amongst adolescent girls constitute a public health concern that has called for immediate evidence-based policy action [9]. In England, only 44% (n = 933) of girls meet Chief Medical Officer Guidelines for physical activity [30].

A variety of factors influence participation, however some of the most frequent barriers identified were a lack of support, including from teachers [9] and menstruation [8]. From our current research, pupils reported PE was affected by menstrual cycle related signs/symptoms. Menstruation was a barrier to participation related to concerns of leaking or use of inserted period products for activities such as swimming. Menstrual cycle related symptoms and dysfunctions were reported to attribute to non-participation. Pupils reported unempathetic teachers towards physical symptoms, specifically menstrual cramps. Other factors such as breast pain, disruption to mood, a lack of motivation and activity type influenced participation in PE. These factors align with those reported by even the most elite female athletes globally, whereby menstrual cramps and other menstrual-related symptoms were reported to negatively affect sport performance [31]. This extends to all participation levels (from recreational to elite) where menstrual-related symptoms compromise exercise participation [32].

However, the education of menstruation and menstrual cycle-related symptom management to remain physically active was absent in the present study. Participants reported no information was provided by teachers or parents on how to manage menstrual cycle-related symptoms, nor was there information pertaining to the benefits of remaining physically active throughout the menstrual cycle and menstruation to support healthy menstrual cycles. This contextualises previously reported figures where 78% of girls who said they used to be ‘sporty’ admitted avoiding taking part in sport when on their period [33]. This issue appears wider than in the United Kingdom. Adolescents in New Zealand receive menstrual cycle education at school but similarly to the United Kingdom, the information they acquire focuses on the biology of the menstrual cycle and neglects how to manage periods during PE [34]. It is essential for teachers to offer alternative exercise to girls when menstruating, reinforcing how exercise can reduce menstrual discomfort [35] through providing appropriate education.

Alongside increased education for pupils, teachers and parents/guardians may benefit from training on the potential impact the menstrual cycle and menstruation has on participation in PE. This should include ways to approach the conversation and ideas for alternative exercise options to increase the likelihood of girls staying active. Teachers are not frequently provided with the training, or possess the expertise, to support girls’; management of menstruation, menstrual cycle-related symptoms or dysfunctions [7]. This is particularly relevant given there is some suggestion that exercise and physical activity may be a prescription for symptom management [e.g. 35].

Teachers, irrespective of their subject disciplines, have previously requested information and support to help deliver and improve confidence/knowledge of the menstrual cycle [7, 36]. This may be delivered to qualified teachers, through continual professional development opportunities, but also through Higher Education teacher training programmes. Resources are emerging [36] to support education. However, resources are required which are specific to the UK curriculum to make this achievable for teachers and educators to ensure time and resource are not barriers for integrating this into schools [7].

### Beyond education in schools

Schools are a significant platform for providing menstrual education, to improve health and wellbeing of pupils across the United Kingdom. However, our current results highlight pupils’ use of the internet as an information source to fill gaps in knowledge and understanding, which may be due to insufficient education. Given the restricted time to provide menstrual education within the school curricula, coupled with limited teacher training to ensure teachers are confident and knowledgeable in their delivery [7], additional evidence-based and high-quality web-based resources could be used to improve menstrual education and management [37].

Within the United Kingdom and beyond, a greater change in society is required. Teachers have described their own personal experiences, facing menarche without adequate support and information. They too have learnt the social and cultural norms of hiding and concealing menstruation [7, 38]. Despite changes reported over time, with age, teachers report the topic was still difficult to share and discuss both in their private life and their role as teachers. Consequently, this has become a barrier to creating a period positive environment [38]. A societal approach to improving knowledge and communication could significantly influence the health of women and girls. Integrating parents into education has previously been effective in the enhancement of menstrual health behaviours, along with peer education interventions [27, 39]. These are two approaches to explore further, alongside providing teacher training to improve knowledge and comfort of communication.

A recent narrative review has concluded to improve overall menstrual health globally, appropriate policies need to be developed which draw upon input from multiple stakeholders to ensure evidence-based and cost-effective practical interventions [40]. Our research highlights the need to ensure girls’ voices are considered when developing menstrual education. The request for lived experiences to be shared was prominent, thus highlighting the potential for people’s stories to be impactful in creating changes both in and outside of school. A recent systematic review supports this, outlining how sharing concerns can give girls confidence and help gain agency of menstrual health [41]. This coincides with the need for education and life-long learning. Fostering a more supportive environment grounded in education can help to overcome barriers [6] and embedding menstrual health literacy into the curriculum is required [40]. It is paramount that females, from an earlier age, maintain physical activity and a general healthy lifestyle. This is not feasible if quality menstrual education is not provided, nor if there is little awareness surrounding the benefits of physical activity for symptom management. For school-aged girls to maintain physical activity levels, key areas for action and policy implementation include an inclusive approach to curriculum development and adequate training of professionals so they have a range of skills to ensure inclusion of adolescent girls in PE [9].

### Limitations

The sample was not representative of the United Kingdom adolescent population. Furthermore, teachers who expressed an interest to participate may bias the results towards schools who actively want to improve menstrual education or have an awareness of the impact. However, the aim was to capture in-depth and rich pupil voice data from a range of locations, including urban and rural, from varying socio-economic status and culture. Conversations were consistent across all locations, but it should be noted this cannot be applied to all situations. It should be acknowledged that the questions focused on general education and further exploration of differences specifically between individuals who have not started menstruating may be beneficial. The sample also included a higher proportion of secondary school pupils, as such, increased voice is required from primary pupils. Additionally, pupil voice was not captured from Scotland or Northern Ireland to infer across the whole of the United Kingdom.

### Conclusions and future research

The abundance of information requested by pupils highlights the deficit in information provided to young people to understand and manage periods in school and specifically in PE. Consideration should be given to lesson adjustments, both in the delivery of menstrual education but also in PE to help girls remain physically active and improve overall health. Schools can utilise information from the current study to create period positive environments. Future research should explore the effectiveness of menstrual education in schools and changes in pupil knowledge. Research highlights involving parents/guardians and peers within menstrual education may be effective approaches and should be explored further. To enable evidence-based information on menstrual-related symptom management, further research is required to understand the cause of symptoms and effectiveness of proactive, lifestyle-based interventions such as physical activity, stress management and diet.

## Data Availability

The anoymised datasets used and/or analysed during the current study are available from the corresponding author on reasonable request. No datasets were generated or analysed during the current study.
